# Changes in Cortisol and Testosterone During Acclimatization and the Relationship to Energetic Outcomes

**DOI:** 10.1002/ajpa.70360

**Published:** 2026-10-10

**Authors:** Mallika S. Sarma, Cara J. Ocobock, Shannon Rochelle, Sarah Martin, Shalender Bhasin, Clare Super, S. Finn Warrick, Lee T. Gettler

**Affiliations:** ^1^ The Health and BioBehavior Laboratory, Department of Anthropology University of Pennsylvania Philadelphia Pennsylvania USA; ^2^ Department of Anthropology University of Notre Dame Notre Dame Indiana USA; ^3^ The Eck Institute for Global Health University of Notre Dame Notre Dame Indiana USA; ^4^ NOLS Rocky Mountain The National Outdoor Leadership School Lander Wyoming USA; ^5^ Research Program in Men's Health, Aging and Metabolism, Boston Claude D. Pepper Older Americans Independence Center Brigham and Women's Hospital, Harvard Medical School Boston Massachusetts USA

**Keywords:** cortisol, energy expenditure, physical activity, sex difference, testosterone

## Abstract

**Objectives:**

When in energetically demanding environments (e.g., high altitude, backcountry expeditions), humans acclimatize to compounded intensive stressors. Acclimatization is possible in part by using the dynamically responsive neuroendocrine system, with changes to hormones like cortisol (CORT) and testosterone (T), which both play important metabolic roles. We sought to examine how these hormones change during acclimatization and what their relationship was to energetic outcomes (i.e., physical activity intensity and average energy expenditure (AEE)).

**Materials and Methods:**

Data were collected from 70 healthy individuals (age range: 18–53 years; female = 39) throughout a 3‐month long expedition in the American Rockies. Repeated measures of pre‐activity salivary baseline and acute change (i.e., reactivity) samples of CORT and T were taken throughout the 3‐month period. Physical activity intensity and energy expenditure were also assessed in a subset of participants using the wGT3X‐BT Actigraph monitor.

**Results:**

Over the 3‐month period, we found that time on the course moderated the relationship between CORT reactivity and physical activity intensity (%MVPA) and AEE. Compared to the start of the expedition, female baseline T was significantly higher in the middle and end, and male baseline T was significantly higher at the end. Additionally, female T acutely declined significantly more at the end of the expedition compared to the beginning. We also found that time on course and sex moderated the relationship between T reactivity and activity intensity.

**Conclusions:**

These findings suggest a dynamic relationship between neuroendocrine and energetic outcomes during acclimatization, which may further differ between sexes.

## 1 Introduction

When exposed to the energetically demanding conditions of high altitude, cold, or resource‐deficient environments for the first time, individuals can experience potent stressors such as hypoxia, thermoregulatory demands, and limited or constrained nutritional and resource availability. Humans respond to these stressors at multiple timescales: while genotypic shifts can take place in populations over generations leading to adaptations shaped by these stressors or phenotypic shifts due to developmental plasticity, even shorter‐term changes also occur due to individual acute acclimatization (Beall 2013; Moore et al. 1998; West‐Eberhard 2003). Acclimatization, where individuals are responding to multiple stressors from the natural world within a relatively shorter timeframe (as soon as ~10 days) (Leonard 2018; Ocobock 2023), is possible in part through physiological changes as the body adjusts to the environment. This includes the dynamically responsive neuroendocrine system, which mediates energy and resource availability in the body in response to external challenges, including ecological and psychosocial pressures. This includes the hypothalamic–pituitary–adrenal (HPA) axis, and its output of cortisol (CORT), and the hypothalamic–pituitary–gonadal (HPG) axis, and its output of testosterone (T) (Ellison 2017; Gettler et al. 2011; Jimeno and Verhulst 2023; Miller and O'Callaghan 2002; Wingfield 2017; Wingfield et al. 1990; Zilioli and Bird 2017). Together, these neuroendocrine shifts both reflect and instigate changes in physiological function and behavior in response to these environmental contexts, which can include variation in physical activity (Brownlee et al. 2005; Sarma et al. 2018, 2020; von Wolff et al. 2018). Beyond the inherent interconnectedness between the HPA and HPG axes, shifts in CORT and T reflect a classic example of trade‐offs between survival/maintenance and reproduction with implications on a variety of outcomes, from energy metabolism to body composition to social behavior (Bribiescas and Ellison 2026; Gettler et al. 2011; Knight et al. 2020; Mennitti et al. 2024; Shirtcliff et al. 2015; Wingfield and Sapolsky 2003).

Less is known about these neuroendocrine changes in response to novel and energetically intensive stressors throughout the acclimatization process, particularly beyond the initial couple of weeks of adjustment. In addition, though CORT and T are tightly linked to energetic outcomes, given the psycho‐physiological nature of the HPA and HPG axes, there is a need to understand which of these changes may be related to specifically physical activity and energy expenditure within this time frame. On expeditions—journeys defined by a specific group of people with a particular purpose—individuals encounter energetically demanding environments, often for the first time, engaging in relatively intensive physical activity, and spending a concentrated amount of time with a small group of other individuals with limited access to resources. Understanding the relationship between neuroendocrine change and energetic outcomes, like physical activity intensity and average energy expenditure (AEE), while acclimatizing to a challenging and novel environment may have important implications for conceptualizing similar processes experienced by our ancestors in the evolutionary past. This aligns with long‐standing evolutionary approaches to reproductive ecology and metabolic physiology (Bribiescas et al. 2012; Ellison 2003, 2017; Jasienska 2003, 2009; Pontzer 2015b; Pontzer et al. 2015). Such insights can also inform our understanding of performance, overall mental and physical health, and even survival in contemporary populations in energetically demanding and precarious conditions.

Neuroendocrine systems play an important mechanistic role in balancing energetic trade‐offs between growth, reproduction, and maintenance based on the demands of an individual's life stage and ecological context (Bribiescas and Ellison 2008; Ellison 2017; Finch and Rose 1995; Kuzawa 2007; Shirtcliff et al. 2015; Worthman and Kuzara 2005). One of the primary functions of the HPA axis, and CORT in particular, is mobilizing physiological resources for energy release after the initial sympathetic nervous system responses, liberating energetic stores directly by mobilizing energy to key tissues (e.g., skeletal muscle, brain), as well as indirectly through behavior (i.e., how time and energy are expended) (Chrousos and Gold 1992; Ice and James 2012; Jimeno and Verhulst 2023; Nelson 2011; Sapolsky et al. 2000; Tsigos and Chrousos 2002). These responses are energetically expensive, and the slightly slower activation of the HPA axis with the release of CORT allows for the necessary energy mobilization. In expedition settings, where individuals are experiencing several novel environmental and social stressors over a set period, CORT response and its related downstream effects (e.g., accelerated heart rate, inhibited digestion, suppressed reproduction) may be critical to individual health and performance when considering a challenging and a continually energetically demanding environment. However, after individuals have acclimatized and are adjusted to their current and relevant stressors (i.e., allostasis), it is possible that the energetic requirements to respond to such a demanding environment may be diminished (Beall 2001; Benso et al. 2007; Kuzawa and Thayer 2011; Pontzer 2015a). In such a context, these diminished energetic requirements may translate to a similarly diminished HPA activation and release of CORT. This lower CORT response as individuals acclimatize may be beneficial to performance and health, since chronically elevated CORT response is linked to negative outcomes across the lifetime such as fatigue and burnout, affective disorders, and medical conditions such as cancer, diabetes, and arthritis (Bower et al. 2005; Ellenbogen et al. 2011; Marchand et al. 2014; Miller et al. 2007).

Conversely, T is dampened and/or suppressed as energy is rerouted for increased energetic needs. Because of its role in costly metabolic functions in males, T production is often acutely suppressed during high levels of energy expenditure in longer term contexts, like endurance activities or subsistence‐lifestyle shifts (Bribiescas 2001; Ellison 2009; Longman et al. 2017). In contrast to these well‐documented effects in males, the comparatively limited research on females hints that they often exhibit increased T during acute intensive physical activity bouts with some links to task performance (Casto et al. 2014, 2020; Casto and Edwards 2016; Ennour‐Idrissi et al. 2015). Androgen production may play an important role in acclimatization effects. While females, like males, produce a range of androgens, a greater percentage of circulating female T derives from the adrenal glands (Longcope 1986), as opposed to primary production by the gonads in males, suggesting adrenal activation potentially accounts for the short‐term elevations in CORT and T among females during energetically costly physical activity. Although little studied in the context demanding physical activity, the peripheral conversion of the adrenal androgen dehydroepiandrosterone can also contribute to local, tissue‐specific synthesis of androgens and estrogens, which could be relevant to energetic allocation. There are also often modest (sometimes stronger) correlations between these other androgens and circulating T and CORT, which vary by sex (Labrie et al. 2001, 2017).

There is more limited research looking at the relationship between energy expenditure, physical activity, and neuroendocrine output in naturalistic, energetically demanding environments. For populations with subsistence‐based lifestyles and/or those indigenous to energetically demanding environments, the relationship between energy expenditure, including aspects of physical activity, and CORT and T output is mixed. Energy expenditure and physical activity intensity were not related to short‐term CORT or T production in Tibetan women living in high altitude regions in Nepal or daily measures of CORT among the Hadza foragers in Tanzania doing daily subsistence tasks (Pontzer et al. 2015; Sarma et al. 2018). Comparatively, in Tsimane forager‐horticulturalists in Bolivia, men maintained relatively elevated CORT during physically intensive and energetically demanding hunting tasks, though only if they were successful in the hunt, and T was positively associated with total energy expenditure (Trumble et al. 2014, 2023). Research among the BaYaka foragers of the Congo Basin demonstrated that women with greater AEE had higher production of CORT throughout the day (Sarma et al. 2020). Though there are methodological differences across these data, these findings potentially support the notion that the relationship between CORT and T production and energetic outcomes, including energy expenditure and physical activity levels, is highly dependent on lifestyle and context of activity, not just the metabolic expense of the activity itself.

Research in non‐subsistence settings has found that individuals in athletic contexts experience acute increases in CORT in response to participation in physically demanding sporting events and exercise tasks (Brownlee et al. 2005; Jürimäe et al. 2011). Comparatively, when individuals are exposed and must acclimatize to energetically demanding environments like high altitude or mountaineering settings, ranger training, etc. over a shorter 6–8 week period, past research demonstrates mixed results: some data show elevated basal CORT and suppressed basal T, whereas others show no change (Benso et al. 2007; Jurimāe et al. 2001; Longman et al. 2017; Mennitti et al. 2024; Nindl et al. 2007; von Wolff et al. 2018). These findings also hint that there is likely nuance in the relationship between intensive physical activity and both CORT and T output given the context of activity when acclimatizing. However, samples in prior work of this nature are often limited by focusing predominantly on males, small sample sizes, cross‐sectional designs, single CORT and/or T measurements over disparate periods, and little attention to neuroendocrine reactivity. Thus, the relationship between energetic outcomes and CORT and T response, specifically as individuals adjust to demanding environments in the shorter acclimatization timescale, is not fully understood.

To help address these questions, we assessed how acclimatization to a novel and energetically demanding environment shaped CORT & T responses and how these responses are related to physical activity intensity and energy expenditure by measuring individuals on expeditions repeatedly over a 3‐month time frame. Working with students participating in National Outdoor Leadership School (NOLS) expeditions through the American Rocky Mountains, we predicted that (1) neuroendocrine profiles would reflect acclimatization, where baseline CORT would be lower and baseline T would be higher at the end of the course compared to earlier in the course, reflecting acclimatization to the stressors and novelty of the environment. Due to CORT's role in energy mobilization for physical activity, we predicted that (2) acute increases in CORT (CORT reactivity) would reflect greater physical activity intensity and energy expenditure; given the elevated costs of being in an energetically demanding environment for the first time, this would be moderated by the length of time on the course. Given the metabolic costs of T, we predicted that (3) controlling for CORT, lower baseline T as well as acute declines in T, suggesting acute suppression of T, would be linked to higher physical activity intensity and energy expenditure but moderated by time on the course. We also hypothesized that the directions of the effects for CORT and T will be similar for males and females, though given that T is more contributory to male reproductive function (and likely) metabolic costs, there may be a greater influence of intensive physical activity and/or energy expenditure for males.

## 2 Methods and Materials

### 2.1 Participants

This study shares the same study design as Sarma et al. (2022) and thus portions of the methods are repeated. In this study healthy individuals (*n* = 70; ages 18–53; see Table 1) took part in one of ten ~90‐day outdoor education courses through NOLS. Participants were part of 10 courses occurring simultaneously between August–December of 2018. Six of these courses were typical fall semester courses, two were designed to train outdoor educators, and two were Wilderness Medicine and Rescue courses. All expeditions were novel to participants: while students had mixed outdoors, backcountry, and general athletic experience, none had participated in an expedition of this duration, and none had engaged in backcountry expeditions in the specific locations selected by NOLS. Of these participants, 31 were male and 39 were female. We recognize there are relevant complexities to a binary reporting of biological sex (DuBois and Shattuck‐Heidorn 2021), so we asked participants to write in self‐identified sex; participants self‐reported as “male” and “female” and are used in the results as such. To participate in a NOLS course, participants had to be generally healthy and willing to comply with a rigorous code of conduct including abstaining from tobacco, alcohol, and illegal substances while on the course. Participants self‐reported medication use which was then coded for contraceptive medication, as this is linked to reproductive steroid production in females, including testosterone (van Anders et al. 2014), and other medications that could be linked with profiles for CORT or T, which we characterized as “hormone‐relevant medication.” Additionally, if relevant, they self‐reported day of last period to calculate menstrual cycle phase at five total time points across the course, corresponding with day of sample collection. Among participants, 72% of females reported menstrual cycle data at some point in the course. Of the data from those participants, 25% of the observations were categorized as missing during at least one point in the course because the participant did not include the information. Please see the supplemental materials for further methodological details and data coding information regarding the medication and menstrual cycle phase variables (see Supporting Information 2). The University of Notre Dame Institutional Review Board approved this study (IRB Protocol 18‐02‐4483) following the US Federal Policy for the Protection of Human Subjects. We recruited from all students taking part in NOLS expedition courses during the fall semester at NOLS Rocky Mountain campus over the age of 18. All persons gave their written informed consent prior to their inclusion in the study.

**TABLE 1:** Descriptives.

Age (mean ± SD)	22.93 ± 6.01 years
Sex	
Males	44.29%
Females	55.71%
Ethnicity	
Asian	4.35%
Native Hawaiian or other Pacific Islander	1.45%
Black or African American	2.90%
White	84.06%
Other	2.90%
Multiple Categories/Mixed	4.35%
Highest education level	
GED	1.45%
High school diploma	36.23%
Some college	31.88%
Associate's degree	1.45%
Bachelor's degree	20.29%
Master's degree	8.70%
Group size (mean ± SD)	13.15 ± 1.9
%MVPA (mean ± SD)	24.10% ± 13.91%
Average EE/h (mean ± SD)	72.68 ± 43.44 kCal/h

### 2.2 Field Settings

NOLS is a US‐based nonprofit organization that teaches leadership and wilderness skills while students live outdoors, often in backcountry settings, for an extended period. Expeditions took place throughout the American West, spanning Wyoming, Utah, Colorado, and Nevada. During the expedition, they experienced altitudes of up to 13,809 ft. and temperatures between −18.9°C and 27.2°C. The typical schedule for a NOLS student would be to arrive in Lander, WY at least 2 days before they leave for their expedition to meet their groups and course leaders and to prepare their gear. Each course has on average 13 students per course, with 3–4 instructors on each section. Each course went through four sections that focused on developing one of the following skills: hiking, mountaineering, canoeing, kayaking, climbing, canyoneering, and winter camping. Each section determined the specific location students were in and what their daily activity would be. Sections were pre‐determined by NOLS Rocky Mountain before courses began and were chosen based on season and following logistical and permitting restraints and had differing expected activity difficulty levels (see Supporting Information Table 1). The wilderness medicine course spent the first section of their course in a shared classroom and field setting becoming certified as wilderness‐emergency medical technicians (wilderness EMT) and were thus excluded from any activity or energy expenditure measurements. In each section, students were required to follow a standardized curriculum where they learned and engaged in the same wilderness and leadership‐related skills and activities.

Data was collected before, after, and at the transitions between sections (referred to as mid‐course checkpoints; see Supporting Information Table 1). We measured body composition, including body mass, lean muscle mass, and percent of body fat (see Table 2) using the Tanita BC‐558 Ironman Segmental Body Composition Monitor bioelectrical impedance scale (Tanita Corporation, Arlington Heights, IL). We used the athletic setting for these measurements due to the increased fitness achieved throughout courses and to maintain measurement consistency. The Tanita equations are unpublished. Measurements were taken before participants consumed a meal at each transition point.

### 2.3 Energy Expenditure and Physical Activity Measurements

Daily AEE (kCal/h) and percentage of time spent in moderate‐to‐vigorous (%MVPA) physical activity measurements were measured over 24 h during the last 10 days of the first section (“early”) and in the last 10 days of the final section (“late”) of the full course in a subset of total participants (*n* = 25). Using the Actigraph wGT3X‐BT monitor, we calculated physical activity levels and AEE using the Freedson VM3 Combination equations using all three axes of movement (*X*, *Y*, *Z*) via “counts” measured through accelerations at 10‐s epochs (Sasaki et al. 2011; see supplemental materials) per 24 h up to 10 days per section, thus creating an AEE value and %MVPA value per day. Time spent in moderate‐to‐vigorous physical activity was calculated via counts per minute cut‐points. The cut‐points used to determine intensity of activity are as follows: light (0–2690 counts per minute), moderate (2691–6166 counts per minute); vigorous (6167–9642 counts per minute); very vigorous (9643–∞) (Sasaki et al. 2011). In this sample, participants only achieved up to moderate activity. Following the Freedson VM3 combination equation, AEE was calculated where if Vector Magnitude Counts per Minute > 2453 kcals/min = 0.001064 × VM + 0.087512(body mass in kg) − 5.500229, else kcals/min = CPM × 0.0000191 × body mass in kg.

### 2.4 Saliva Samples

Saliva samples were collected across three time points on the last day of each section across four sections: (1) pre‐activity (9:47 AM ±186 min; obs = 189), (2) mid‐activity (10:59 AM ±190 min; obs = 187), and (3) post‐activity (15:03 PM ±222 min; obs = 175). Samples were self‐collected via passive drool and placed in plastic bags with icepacks until they could be transported to liquid nitrogen dewars. Samples were kept cold with icepacks for 1–2 days before they were transported to frozen conditions. Individuals were not allowed to use tobacco while participating on the course and were asked to rinse out their mouths before providing saliva samples.

Samples were frozen and stored on site in portable liquid nitrogen dewars and were shipped to the Hormones, Health, and Human Behavior Lab at the University of Notre Dame on dry ice where they were stored at −80°C. Prior to assay procedures, saliva samples were visually inspected for discoloration (such as from blood or food contamination). Samples were vortexed and centrifuged at 3000 rpm for 15 min to help remove particulate matter (such as from food) from the saliva. In all, 27 samples were excluded from analysis due to lack of required volume or blood contamination and are not included in the reported numbers above.

### 2.5 Salivary Cortisol

We treated the first sample taken before activity on the last day of each section as *baseline CORT*. All baseline CORT values at each collection point were base‐ log_10_ transformed for normality. CORT reactivity was calculated as the percentage. change between pre‐activity and mid‐activity raw CORT samples within the first hour; this time frame is the window during which CORT reactivity is well documented to rapidly responding to physical and psychosocial demands (Dickerson and Kemeny 2004; Edwards and Casto 2013). To stay within this relevant time frame, reactivity values were calculated only using samples that were within the first hour of overall activity. For these values, there was on average 36 ± 7.8 min between pre‐activity and mid‐activity (see Figure 1).

Concentrations of CORT were determined using commercially available kits for enzyme immunoassay protocol developed for saliva samples (Salimetrics, Carlsbad, CA; Kit No. 1‐3002). Samples were assayed in duplicate by MSS. CORT inter‐assay coefficients of variation were 4.1% and 5.7% for high and low kit‐based control samples respectively; the intra‐assay CV for CORT was 6.0%.

### 2.6 Salivary Testosterone

Similarly, baseline T was from the first sample taken before activity and values were base‐ log_10_ transformed for normality. To look at T across intensive activity, we used pre‐activity and mid‐activity raw T samples within the first hour. For these values, there was on average 36 ± 8.15 min between pre‐activity and mid‐activity (see Figure 1).

**TABLE 2:** Anthropometrics and baseline and reactivity endocrine values across the course by sex (mean ± SD).

	Section 1		Section 2		Section 3		Section 4	
	Female	Male	Female	Male	Female	Male	Female	Male
Body mass (kg)	62.53 ± 9.56 kg	77.42 ± 9.75 kg	64.35 ± 9.02 kg	75.49 ± 17.67 kg	63.21 ± 6.72 kg	78.29 ± 9.94 kg	63.83 ± 8.57 kg	76.76 ± 10.25 kg
Body fat (%)	22.38% ± 6.89%	12.28% ± 4.53%	24.04% ± 6.21%	12.99% ± 3.43%	23.66% ± 6.32%	12.82% ± 4.22%	22.85% ± 6.41%	11.88% ± 2.56%
Lean muscle mass (kg)	46.15 ± 4.32 kg	62.77 ± 12.03 kg	45.66 ± 9.02 kg	60.25 ± 18.29 kg	44.06 ± 10.99 kg	62.53 ± 14.54 kg	46.35 ± 3.49 kg	65.03 ± 10.17 kg
Baseline CORT (μg/dL)^a^	0.28 ± 0.15 μg/dL	0.30 ± 0.19 μg/dL	0.30 ± 0.23 μg/dL	0.23 ± 0.22 μg/dL	0.34 ± 0.38 μg/dL	0.29 ± 0.22 μg/dL	0.36 ± 0.24 μg/dL	0.35 ± 0.26 μg/dL
CORT reactivity (% change)^a^	−13.39% ± 52.34%	−27.84% ± 35.50%	−9.91% ± 27.71%	3.03% ± 80.49%	−8.33% ± 32.97%	−4.33% ± 45.74%	−4.70% ± 38.85%	−19.90% ± 25.36%
Baseline T (pg/mL)^b^	6.39 ± 10.78 pg/dL	105.55 ± 51.38 pg/dL	14.51 ± 21.15 pg/dL	108.30 ± 44.60 pg/dL	8.74 ± 15.58 pg/dL	126.91 ± 68.28 pg/dL	8.13 ± 11.30 pg/dL	152.06 ± 75.26 pg/dL
T reactivity (% change)^b^	273.27% ± 497.86%	−28.23% ± 25.43%	33.30% ± 147.36%	−3.06% ± 18.85%	175.74% ± 341.87%	1.32% ± 26.08%	10.89% ± 132.99%	1.57% ± 29.26%

^a^ Analyzed via ELISA.

^b^ Analyzed via LC–MS/MS.

**FIGURE 1:**
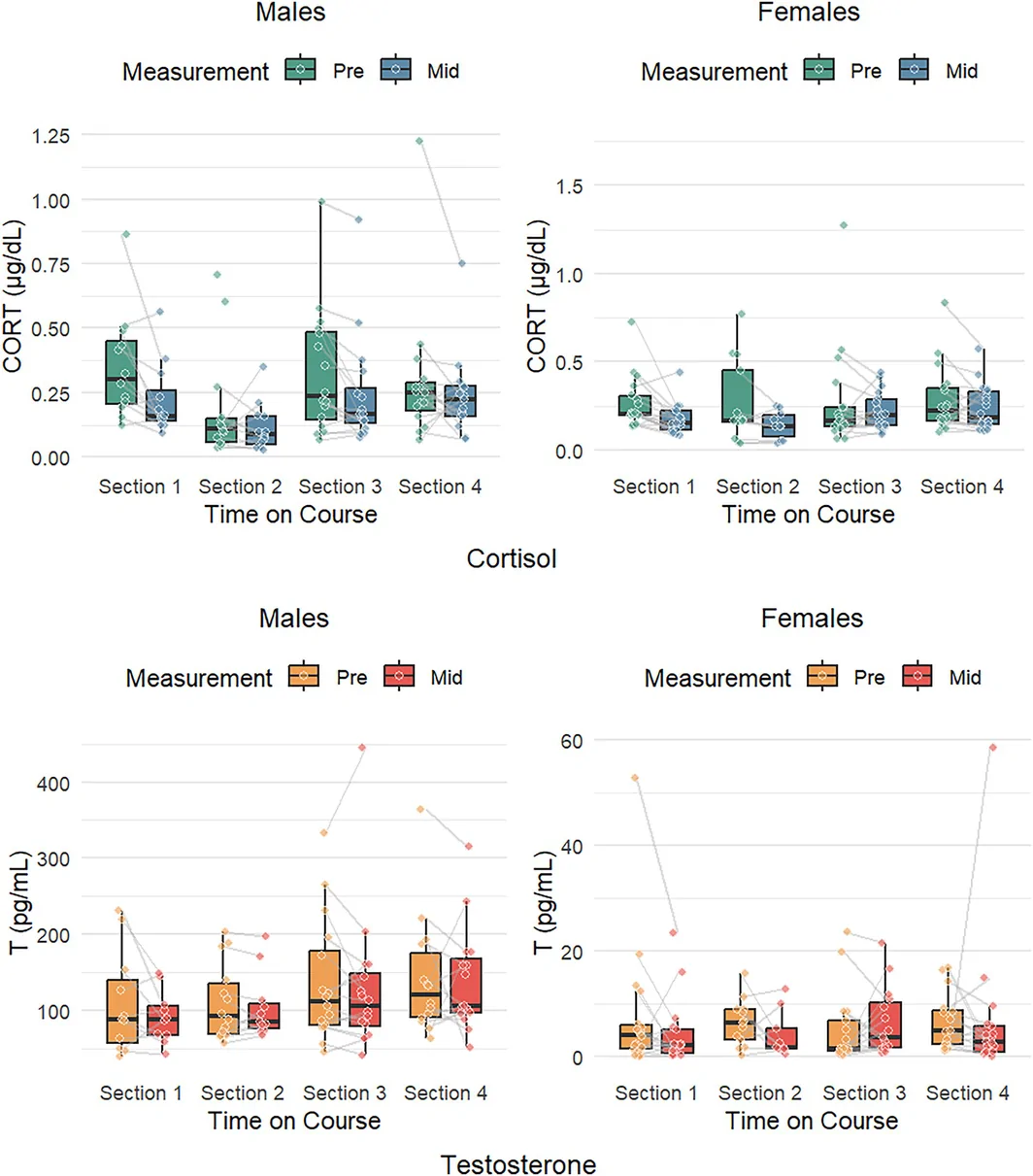
Boxplots of baseline and mid‐activity CORT & T by time on course. Raw values for CORT and T for “pre” (i.e., baseline) and “mid” (i.e., mid‐activity) values for each section of the course. See Table 2 for mean CORT and T baseline and reactivity values. Bars represent the interquartile range, with error bars indicating the range of CORT and T outcomes, respectively. While there was no significant difference in either baseline CORT or CORT reactivity (percentage change between mid‐activity and baseline CORT), when adjusting for relevant covariates, we found that baseline T was higher in the second and 4th section of the course, and T reactivity had a greater acute decline in the 4th section of the course compared to the first section of the course (all *p* < 0.05). See Table 2 for raw baseline and reactivity values and Table 3a for T values across the course. See Supporting Information Table 1 for CORT values across the course.

Concentrations of T in males were determined using commercially available kits for enzyme immunoassay protocol developed for saliva samples (Salimetrics, Carlsbad, CA; Kit No. 1‐2402) and samples were assayed in duplicate by Salimetrics. As reported by Salimetrics, T average inter‐assay coefficients of variation for this analysis were 9.9%; the intra‐assay CV for T was 4.6%. Given that female T often is at the very lowest limits of detection for ELISA analyses, salivary testosterone concentration in female participants was measured in the Brigham Research Assay Core Laboratory (Shalender Bhasin, Director), using a highly sensitive and specific Liquid Chromatography–Tandem Mass Spectrometry (LC–MS/MS) assay, certified by the Centers for Disease Control and Prevention's Hormone Standardization Program for Testosterone (HoST); the assay details have been published (Huang et al. 2022; Jasuja et al. 2023; Thurston et al. 2018). Briefly, testosterone in human saliva was extracted by solid phase extraction, separated by high performance liquid chromatography (HPLC), and measured by mass spectrometry (MS) in electrospray ionization (ESI+) source. Deuterated testosterone (16,16, 17‐D3 testosterone) was added to each sample as an internal standard. The lower limit of quantitation was 5 pg/dL. The cross‐reactivity of DHEA, DHEAS, DHT, androstenedione and estradiol in the testosterone assay is negligible at ten times the circulating concentrations of these hormones. The inter‐assay CV was < 5% for the NIST female standard 971 and < 2% for NIST male standard 971. As part of the Centers for Disease Control and Prevention's Hormone Standardization Program for Testosterone (HoST), quality control samples provided by the HoST were run every 3 months. The mean bias was −0.5% (−2.7 to +1.7%) in the female samples and −0.9% (−2.6 to +0.3%) in the male samples. The imprecision (CV%) was 2.9% in the female samples and 1.9% in the male samples.

### 2.7 Statistical Analysis

All analyses were run in Stata version 19 (Stata Corporation, College Station, TX) and figures were made in both Stata and R (R Core Team, 2025). CORT and T models were divided by sex, given the likely magnitude of difference in neuroendocrine values as well as the different methods of analysis for T samples. We used linear mixed models to estimate change in baseline CORT and T, respectively, and %change in CORT and T, respectively, over time, adjusting for age, body fat. Additionally, following the NOLS predetermined section schedule, we also adjusted for a dichotomous physical difficulty categorization (i.e., “high physical difficulty” vs. “low physical difficulty”) reflecting NOLS programmatic structure of each section (see Supporting Information Table 1). For baseline CORT and T models, we also included medication use as a covariate (see below).

We then used linear mixed models to estimate if the interaction of %MVPA and AEE, respectively, with time on the course and sex together predicted baseline CORT and CORT reactivity and predicted baseline T and T reactivity adjusting for age, and body fat. These models were combined by sex to allow for a larger sample size in each model. As such, for T models, given the differences in laboratory analysis (i.e., ELISA for male samples and LC–MS/MS for female samples), outcome values were transformed into z‐scores to combine male and female data and reduce the likelihood of Type 2 error. Given that both %MVPA and AEE had up to 10 days of data for each collection point, but CORT and T values were from a single collection point, the energetic variables were treated as the dependent variable, and endocrine values were replicated and matched to each day of energetic data. All baseline CORT and T models were adjusted for their baseline (pre‐activity) sampling times, respectively. CORT and T reactivity models were further adjusted for mid‐activity sampling time and the length of time between pre‐activity and mid‐activity samples. All T models also were adjusted for baseline CORT. While all raw hormonal values used in the models were within the range of biological viability, in reactivity models %change values greater than 8 SD over the mean were excluded as biologically implausible.

In addition, given that contraceptive medication use (for females), hormone‐relevant medication use (for males), and menstrual cycle phase (for females) were potentially pertinent covariates to our CORT and T variables, we evaluated their fit in two ways. First, we tested whether each of these three variables, respectively, was associated with the CORT and T variables (see Supporting Information Tables 2–5). Second, we also used Bayesian Information Criterion (BIC) analyses to assess the model fit if these variables were included as covariates in our main models. In our initial analyses, in independent models, contraceptive medication use and hormone‐relevant medication use, respectively, were associated with baseline CORT and T outcomes, though the 95% CIs included zero. When we included these variables as covariates in our main models predicting baseline CORT and T as outcomes, the model fit (assessed via BIC) was not meaningfully changed (< 2 BIC difference). Thus, we included contraceptive medication use and hormonal‐impacting medication use as covariates in the baseline CORT and T models. Neither medication‐use variable significantly or meaningfully predicted CORT or T reactivity outcomes, and their inclusion in the pertinent main reactivity models led to substantially poorer model fit (via BIC). Thus, we excluded them to avoid overfitting. Menstrual cycle phase did not significantly or meaningfully predict any hormonal outcomes, did not improve model fit for our main models, and involved loss of observations due to missing data. Thus, we excluded this from our main models (see Supporting Information Tables 4 and 5). We evaluated statistical significance at *p* < 0.05.

## 3 Results

### 3.1 Change in CORT Across the Course

Using linear mixed models and adjusting for relevant covariates, we found that course section did not significantly predict baseline CORT or CORT reactivity in males or females (see Figure 1 and Supporting Information Table 2).

### 3.2 Change in T Across the Course

In equivalent models of T, we found that male baseline T was significantly higher during the last section of the course (*b* = 0.18, 95% CI [0.01, 0.46], *p* = 0.044) compared to the first section (see Table 3a), adjusting for relevant covariates, including baseline CORT. We also found that female baseline T was significantly higher during the second section (*b* = 1.12, 95% CI [0.15, 2.09], *p* = 0.024) and the fourth (last) section of the course (*b* = 0.79, 95% CI [−0.06, 1.50], *p* = 0.033) compared to the first section (see Table 3a), adjusting for relevant covariates, including baseline CORT. We also found in females that T reactivity was significantly different in the last section of the course, relative to the first section (*b* = −307.99, 95% CI [−548.44, −67.55], *p* = 0.012; see Table 3b). On average, females exhibited substantially larger declines in T reactivity in the last section compared to the first section. Comparatively, we found that course section did not significantly predict baseline T or T reactivity in males (all *p* > 0.06; Table 3b).

**TABLE 3a:** Linear mixed models predicting baseline T from time on course.

	Baseline logged T^d^					
	Male^a^			Female^b^		
	*b*	95% CI	*p*	*b*	95% CI	*p*
Main effects						
Timepoint						
Section 2	0.18	−0.13, 0.48	0.252	**1.12**	**0.15, 2.09**	**0.024**
Section 3	0.10	−0.16, 0.37	0.443	0.37	−0.43, 1.18	0.360
Section 4	**0.23**	**0.01, 0.46**	**0.044**	**0.79**	**0.06, 1.50**	**0.033**
Covariates						
Logged baseline CORT	**0.26**	**0.12, 0.41**	**0.000**	−0.003	−0.43, 0.43	0.990
Baseline sampling time	−0.02	−0.06, 0.02	0.334	0.005	−0.10, 0.11	0.928
Age	−0.02	−0.02, 0.003	0.142	−0.05	−0.10, 0.01	0.084
Body fat	**4.33**	**1.92, 6.74**	**0.000**	−3.89	−8.94, 1.17	0.132
Section difficulty	−0.08	−0.32, 0.16	0.528	−0.09	−0.89, 0.70	0.823
Medications^c^	**−0.22**	**−0.43, −0.01**	**0.042**	**−0.69**	**−1.34, −0.04**	**0.039**

^a^ obs = 75.

^b^ obs = 93.

^c^ Male medications include all hormone‐relevant medication; female medications are specifically contraceptive medications.

^d^ Bolded values represent *p* < 0.05.

**TABLE 3b:** Linear mixed models predicting T reactivity from time on course.

	T reactivity^c^					
	Male^a^			Female^b^		
	*b*	95% CI	*p*	*b*	95% CI	*p*
Main effects						
Timepoint						
Section 2	9.25	−24.99, 43.49	0.596	−216.42	−602.77, 169.93	0.272
Section 3	22.50	−5.29, 50.30	0.113	−109.26	−393.05, 174.52	0.450
Section 4	22.68	−2.60, 47.96	0.079	**−307.99**	**−548.44, −67.55**	**0.012**
Covariates						
Logged baseline CORT	−7.47	−20.71, 5.76	0.269	−20.09	−146.06, 105.89	0.755
Reactivity sampling time	−1.26	−4.61, 2.08	0.459	−4.39	−31.13, 22.36	0.748
Reactivity time range	15.70	−32.53, 63.93	0.523	−49.48	−495.89, 396.93	0.828
Age	−0.30	−1.44, 0.84	0.608	7.41	−4.66, 19.48	0.229
Body fat	**261.11**	**76.40, 445.82**	**0.006**	749.03	−427.57, 1925.64	0.212
Section difficulty	−7.19	−30.86, 16.49	0.552	94.47	−174.15, 363.10	0.491

^a^ obs = 40.

^b^ obs = 56.

^c^ Bolded values represent *p* < 0.05.

### 3.3 Physical Activity, AEE, and CORT

For baseline CORT, neither the conditional effects nor the interaction effects of baseline CORT, sex, and time on the course predicted %MVPA or kCal/h. In models focusing on CORT reactivity, we found that the interaction of CORT reactivity with time on the course significantly predicted %MVPA (*b* = −0.002, 95% CI [−0.004, −0.0003], *p* = 0.027) and average kCal/h (*b* = −0.65, 95% CI [−1.27, −0.02], *p* = 0.042; see Table 4, Figure 2, and Figure 3).

**TABLE 4:** Linear mixed models predicting %MVPA and AEE from CORT reactivity and the moderating effects of time on course and sex.

^c^	%MVPA^a^			AEE (kCal/h)^b^		
	*b*	95% CI	*p*	*b*	95% CI	*p*
Conditional effects						
% change CORT	0.001	−0.00017, 0.0018	0.102	0.14	−0.16, 0.44	0.351
Timepoint (last section compared to first section)	−0.02	−0.10, 0.054	0.536	−8.63	−31.96, 14.71	0.469
Sex	0.14	−0.21, 0.49	0.434	14.15	−83.37, 111.66	0.776
Interactions						
% change CORT × timepoint	**−0.002**	**−0.0045, −0.000027**	**0.027**	**−0.65**	**−1.27, −0.02**	**0.042**
Timepoint × sex (male)	−0.15	−0.50, 0.19	0.377	13.42	−82.72, 109.55	0.784
Sex (male) × % change CORT	0.003	−0.0035, 0.01	0.344	−0.01	−1.94, 1.92	0.991
% change CORT × timepoint × sex (male)	−0.002	−0.0086, 0.0055	0.661	0.46	−1.58, 2.49	0.661
Covariates						
Reactivity sampling time	−0.01	−0.044, 0.016	0.375	−2.39	−9.79, 5.01	0.527
Reactivity time range	**−0.19**	**−0.37, −0.0015**	**0.033**	**−71.28**	**−122.01, −20.55**	**0.006**
Age	0.01	−0.0032, 0.017	0.183	1.580	−1.00, 4.16	0.231
Body fat	**−0.66**	**−1.30, −0.027**	**0.041**	−38.67	−222.95, 145.62	0.681

^a^ obs = 211.

^b^ obs = 211.

^c^ Bolded values represent *p* < 0.05.

**FIGURE 2:**
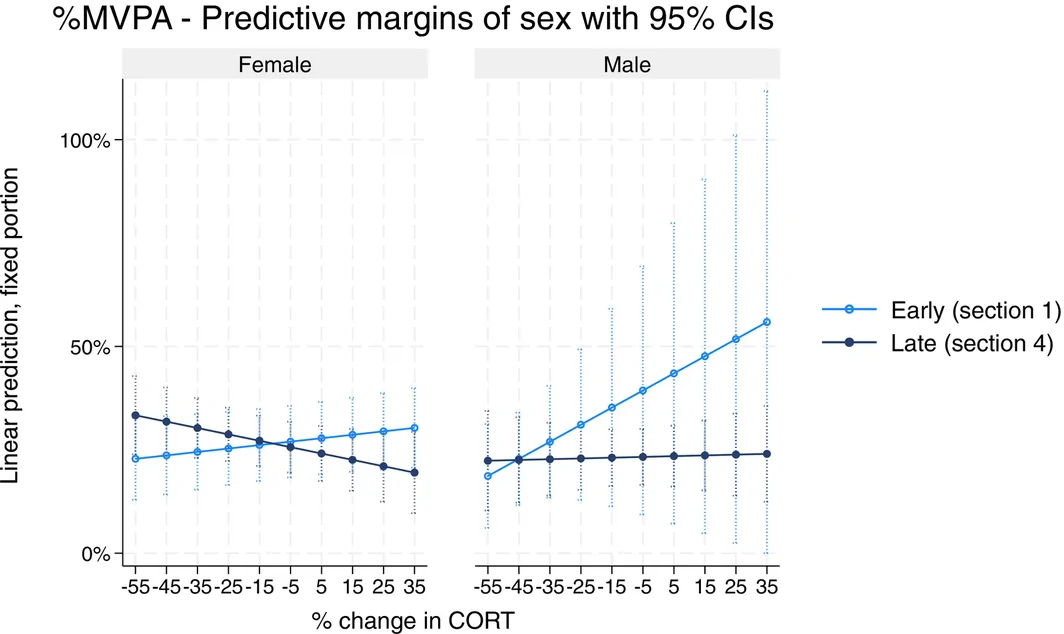
Interaction model of CORT Reactivity predicting %MVPA early and late in the course, by sex. In the early section of the course, there was a positive relationship between CORT reactivity and %MVPA, where acute spikes in CORT were related to greater physical activity intensity. In the final section of the course, there was a negative relationship between CORT reactivity and %MVPA, where greater acute spikes in CORT predicted lower physical activity (*p* < 0.05). There is no clear effect of sex. See Table 4 for the linear mixed model. The lines represent slopes early and late in the course estimating the fixed portion in the linear prediction for a linear mixed model.

**FIGURE 3:**
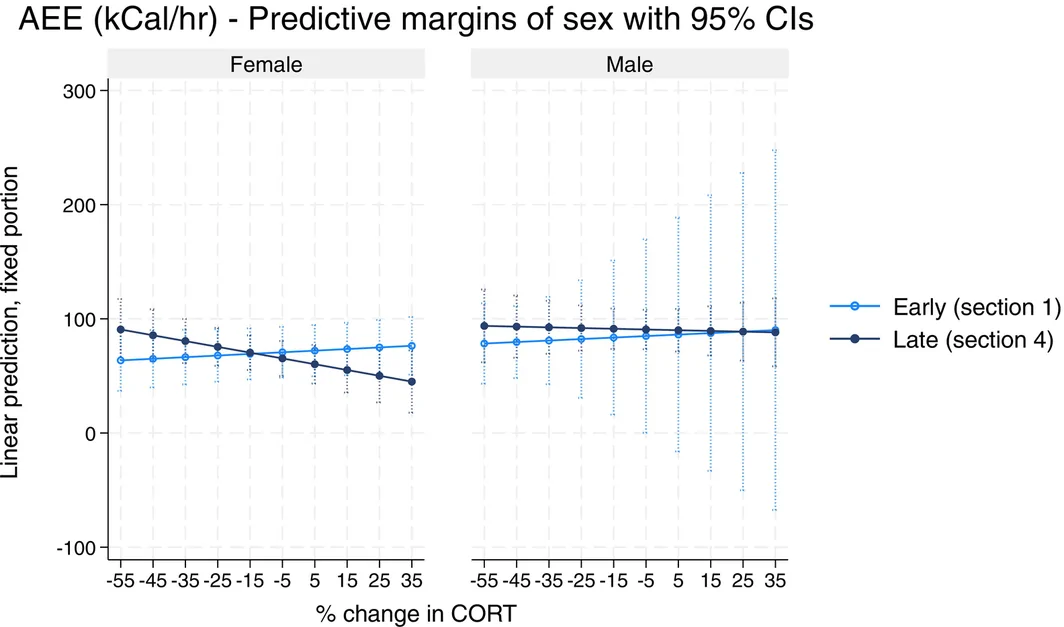
Interaction model of CORT Reactivity predicting AEE early and late in the course, by sex. In the early section of the course, there was a positive relationship between CORT reactivity and AEE, where acute spikes in CORT were related to greater average kCal/h. In the final section of the course, there was a negative relationship between CORT reactivity and AEE, where greater acute spikes in CORT predicted lower average energy expenditure (*p* < 0.05). There is no clear effect of sex. See Table 4 for the linear mixed model. The lines represent slopes early and late in the course estimating the fixed portion in the linear prediction for a linear mixed model.

### 3.4 Physical Activity, AEE, and T

We found that neither the conditional effects nor the interactions of baseline T, sex, and time on the course significantly predicted %MVPA or kCal/h. In models looking at T reactivity, we found that the interaction of T reactivity and time on course (*b* = −0.001, 95% CI [−0.001, −0.00002], *p* = 0.042) and the interaction of T reactivity, time on course, and sex (*b* = −0.01, 95% CI [−0.0008, −0.012], *p* = 0.025) predicted %MVPA (see Table 5 and Figure 4). Neither the conditional effects nor the interactions of T reactivity, sex, and time significantly predicted AEE.

**TABLE 5:** Predicting %MVPA and EE from T reactivity and the moderating effects of time on course and sex.

^c^	%MVPA^a^			AEE (kCal/h)^b^		
	*b*	95% CI	*p*	*b*	95% CI	*p*
Conditional effects						
% change T	0.0001	−0.10, 0.28	0.535	−0.01	−0.08, 0.05	0.675
Timepoint (last section compared to first section)	0.09	−0.17, 0.06	0.337	−0.59	−58.81, 57.63	0.984
Sex (male)	−0.06	−0.17, 0.06	0.330	2.61	−35.58, 40.81	0.893
Interactions						
% change T × timepoint	**−0.001**	**−0.00014, 0.00026**	**0.042**	−0.13	−0.34, 0.069	0.196
Timepoint × sex (male)	0.03	−0.13, 0.19	0.739	31.85	−19.88, 83.58	0.228
Sex (male) × % change T	−0.005	−0.01, 0.001	0.111	−0.47	−2.06, 1.11	0.561
% change T × timepoint × sex (male)	**−0.01**	**0.0008, 0.012**	**0.025**	0.70	−0.93, 2.34	0.400
Covariates						
Logged baseline CORT	**0.08**	**0.018, 0.14**	**0.011**	13.51	−6.85, 33.88	0.193
Reactivity sampling time	0.02	−0.0074, 0.05	0.138	5.65	−1.61, 12.90	0.127
Reactivity time range	**−0.27**	**−0.43, −0.098**	**0.002**	**−61.60**	**−110.71, −12.50**	**0.014**
Age	−0.002	−0.01, 0.01	0.716	0.36	−2.16, 2.89	0.778
Body fat	−0.68	−1.52, 0.15	0.109	66.61	−196.45, 329.68	0.620

^a^ obs = 188.

^b^ obs = 188.

^c^ Bolded values represent *p* < 0.05.

**FIGURE 4:**
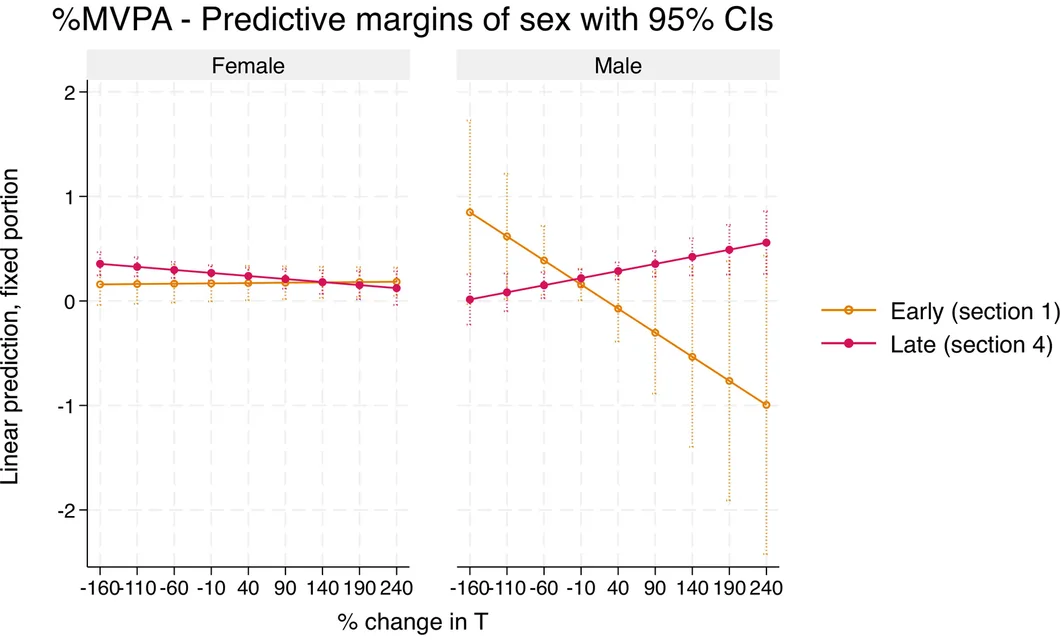
Interaction model of T Reactivity predicting %MVPA early and late in the course, by sex. Early in the course, males showed greater acute declines in T with higher activity intensity and larger spikes in T with lower activity intensity. In contrast, later in the course, males exhibited greater acute declines in T with lower activity intensity and acute spikes in T with higher activity intensity (*p* < 0.05). See Table 5 for the linear mixed model. The lines represent slopes early and late in the course estimating the fixed portion in the linear prediction for a linear mixed model.

## 4 Discussion

In a novel and energetically demanding environment, like backcountry expeditions, stressors experienced in the environment are broad and can encompass a range of stimuli from hypoxia to shifts in magnitude and/or kind of physical activity to interacting with new social groups. Compared to acclimating to a single stressor, which is often the focus of study designs conducted in controlled laboratory settings, acclimatization occurs in response to the cumulative effect of these variable stressors in natural world field conditions (Leonard 2018) and can be seen in an expedition setting. Here, we demonstrate evidence supporting such acclimatization through both the fluctuation in energetically linked hormones (i.e., CORT and T) and the changing dynamics between acute endocrine reactivity and energetic outcomes (i.e., physical activity intensity and energy expenditure). These data highlight the complex physiological response to novel and demanding environmental stressors, pointing to acclimatization processes that lie along a greater “human adaptation continuum” with responses ranging from seconds to hundreds of thousands of years (Kuzawa and Pike 2005; Kuzawa and Thayer 2011).

### 4.1 Dynamics of Baseline CORT and CORT Reactivity and Relationship to Energetic Outcomes

Previous studies using single measurements of CORT before and after expeditions have found mixed outcomes for CORT output, including reduced CORT at the end compared to the beginning or no change to CORT at all (Benso et al. 2007; Farrace and Cenni 1999). Since less work has explored CORT changes during the acclimatization process, we hypothesized that acclimatization would be reflected via changes to baseline CORT at different time points on the course. However, we found that time on the course, at any checkpoint, did not significantly predict either baseline CORT or CORT reactivity for NOLS participants. Although caution is warranted when interpreting null results, this potentially suggests that across the 3‐month period, CORT output may not shift to new set points, and instead it remains relatively consistent while other physiological measures fluctuate. Alternatively, it could be that the expedition experience is not enough of a stimulus for change in CORT output. Feedback about the courses suggests an acutely intensive experience often reported by participants (Personal Communication Aug‐Dec, 2018; Froehly et al. 2025; Jostad et al. 2012; Sibthorp et al. 2007). However, our earlier published work with this group has shown that while body fat declined, body weight rebounded throughout the expedition and thus indicates that participants were unlikely to be in a negative energy balance (Sarma et al. 2022), on average, which helps mitigate one possible and major stressor.

Comparatively, supporting our hypothesis that acute increases in CORT (i.e., CORT reactivity) would predict greater activity intensity and AEE, we did find that the interaction of CORT reactivity and timepoint significantly predicted both %MVPA and AEE (kCal/h). Specifically, we found that in the early section of the course, there was a positive relationship between CORT reactivity and %MVPA and average kCal/h, where acute spikes in CORT were related to both greater physical activity intensity and greater daily AEE. Interestingly, in the final section of the course, there was a negative relationship between CORT reactivity and %MVPA and AEE, where greater acute spikes in CORT predicted lower physical activity intensity and lower average kCal/h.

The shifting relationship between CORT reactivity and both activity intensity and energy expenditure in females between the first and last section suggests a potential acclimatization effect. This is in line with the role of CORT as a primary mechanism for energy mobilization in response to physical activity that is highly responsive and sensitive to experience and intensity (Brownlee et al. 2005; Budde et al. 2015; Gatti and De Palo 2011; Hill et al. 2008). Here, we posit that initially, with more time spent in more intensive physical activity, CORT output would be higher to mobilize enough energy necessary early in the course. Over time, as individuals acclimatize to the energy intensive environment, acute increases in CORT do less to mobilize energy stores for physical activity and general energy expenditure and may instead be elevated due to other demands, like psychosocial demands. Unlike in more controlled experimental settings (Alghadir et al. 2015; Brownlee et al. 2005), we do not find a consistent relationship between greater CORT and increased energy expenditure across the entire period. One possibility is that in field conditions, compared to a controlled lab environment, there are additional factors (i.e., social evaluation threat, unpredictability, etc.) that create a more dynamic relationship between CORT and its role of releasing and mobilizing energy, particularly in an energetically intensive setting (Dickerson and Kemeny 2004; Flinn et al. 1998; Saxbe 2008). In addition, given the well‐characterized psychobiological roles of CORT, there is potentially a cognitive component of intense effort related to fear, anticipation, etc. that is linked to HPA axis and CORT response (Carroll et al. 2017; James et al. 2023; Russell and Lightman 2019) but may fail to be captured by accelerometry‐specific measurement of AEE.

### 4.2 baseline t and T Reactivity Dynamics and Relationship to Energetic Outcomes

It is well documented that intensive exercise over a longer period of time suppresses sex hormones in both men and women (Ennour‐Idrissi et al. 2015; Feicht et al. 1978; Mennitti et al. 2024; Wheeler et al. 1984), though the specific hormones and magnitude of suppression may differ between sexes. However, this suggests that the demands of energetically intensive environments would likewise suppress a sex hormone like T. In longer term exposures to such environments, where acclimatization is anticipated, there is evidence of declines in baseline T (Benso et al. 2007; Jimenez et al. 2019; Nindl et al. 2007; Pivarnik et al. 1987). However, as discussed earlier, most of these studies only consider single measurements of T, use small sample sizes, and typically include only male participants. T reactivity in this context, both in males and females, has been much less studied.

We found some similarity in male and female patterns of baseline T across the course. We found that baseline T in both males and females significantly increased in the last section of the course in comparison to the first section. Similarly, females' baseline T also was significantly higher in the second section of the course compared to the first section. In contrast, female T acutely declined significantly more in the final section of the course compared to their T reactivity during the first section. We did not find shifts in male T reactivity across the course. Given that we adjusted these analyses for activity difficulty, these patterns suggest that time spent on the course (i.e., acclimatizing) does play an important role in T outcomes. Specifically, as individuals acclimatize to the environment, energetic constraints may be lifted to allow for a higher baseline T. In this sample, we see this possible acclimatization response via increase in T in the final section of the course and additionally in females, during the second section of the course, towards the middle of the full expedition. For T reactivity, females showed significantly larger acute decline in T after activity, which may suggest a more calibrated response to physical activity, indicating reduced production of a hormone that can increase energetic costs. Since we cannot tease apart sex and gender in this sample, it is possible that the differences between specific sections reflect social gendered behavior that happen to overlap with sex‐specific patterns. For example, given the immersive sociality of the expedition, one possibility is that these significant female T changes are a responding to gendered behaviors and/or perceptions of stereotyped behavior related to other dynamics of social relationships and interactions (Casto et al. 2014; Kornienko et al. 2016; van Anders et al. 2015, 2014; van Anders and Watson 2006). Though there is limited research on gendered behaviors in intensive backcountry or expeditionary settings, work has suggested that men and women behave and perceive experience differently in mixed‐sex groups (de Souza and Junior 2024; Ouellet and Laberge 2023), potentially influencing neuroendocrine outputs sensitive to social demands and activity. Alternatively, or perhaps additionally, it is possible that changes in T are related to thermoregulation. Research has demonstrated that sex hormones, and T specifically, are linked in thermoregulation and cold sensitivity in mammals, including humans (Fernández‐Peña et al. 2023; Gkika et al. 2020; Monjo et al. 2003). In these backcountry environments specifically, temperature variation can be substantial, ranging from −26.8°C to 46.7°C (Ocobock 2016a), where increased thermoregulatory demands are likely. The measures of both physical activity intensity and energy expenditure are accelerometry‐based and thus might exclude thermoregulatory demands.

Aligning with our hypothesis, there was a difference in male and female outcomes, especially as related to energetic outcomes. The interaction of T reactivity and time on course predicted activity intensity; additionally, the interaction of T reactivity, time on course, and sex also predicted activity intensity. These findings suggest differential acclimatization patterns between males and females. Early in the course, males showed greater acute declines in T with higher activity intensity and larger spikes in T with lower activity intensity. In contrast, later in the course, males exhibited greater acute declines in T with lower activity intensity and acute spikes in T with higher activity intensity. In all, this supports our hypothesis of an acclimatization effect throughout the exposure period of the expedition. It further suggests that these acclimatization patterns related to activity intensity might be sex specific. This aligns with our understanding of reproductive hormone suppression during prolonged, intensive physical activity, particularly since T plays a more central critical role to reproductive function in males, with a much larger percentage of circulating levels being produced by the gonads, compared to females (Bribiescas 2001; Gettler et al. 2010).

In all, significant neuroendocrine changes and a shifting relationship between CORT and T with activity intensity and average EE suggest acclimatization, though likely through complex mechanisms. These findings demonstrate a dynamic relationship between neuroendocrine and energetic outcomes, where neuroendocrine reactivity as well as the specific relationship between immediate changes in CORT and T (i.e., reactivity) and energetic outcomes may both change over exposure time, and specific to reproductive hormones like T, may differ between sexes.

There are several limitations with these data that merit discussion. First, though we were able to collect data from a relatively large sample size for this type of intensive context, given the number of measurements collected in the study, the challenging conditions, and the process of self‐collection, there was some variation participants' ability to complete all components of the study at the same time. Given the close relationship the focal hormones have with circadian rhythms, this variability may have impacted our findings. To best correct for this fluctuation all models account for time of collection. In future studies, we recommend time of collection along with measurement of waking time (e.g., using accelerometers that distinguish waking periods with accuracy and precision when worn for 24‐h periods) to better anchor the hormonal data (Gettler et al. 2023). Additionally, similar work could benefit from tracking sleep (including quality and quantity), which interrelates with the production and functions of CORT and T (Gettler et al. 2023; Lateef and Akintubosun 2020; O'Byrne et al. 2021). Our statistical power was limited due to the smaller sample size of actigraphy data that were a subset of the larger study population. However, we compensated for this by drawing on repeated, day‐by‐day accelerometry data to increase observation number for the actigraphy‐based analyses. Accelerometry is a standard field‐based estimate based on activity counts and acceleration rather than actual direct total EE; however, because it is an estimate, we may be failing to fully capture EE. Collecting AEE via actigraphy would have been augmented by also collecting this same data via doubly labeled water (DLW), that is, the gold‐standard of measuring total energy expenditure (Ocobock 2016b). Additionally, while research linking CORT to menstrual cycle phase is mixed, particularly in individuals not naturally cycling due to contraceptives, T is known to increase across the days of the ovulatory phase with differential impact during intensive physical activity (Huang et al. 2022; Kirschbaum et al. 1999; Klusmann et al. 2023; Kudielka and Kirschbaum 2003; van Anders et al. 2014). Though our supplementary analyses here do not suggest a significant or meaningful relationship between menstrual cycle phase and female participants' T and CORT profiles in this, we cannot fully rule out its potential impact. For the laboratory analysis, we analyzed all CORT samples using ELISA. For T samples, we analyzed male T using ELISA. However, because female T values are lower, on average, and are often near the to lower limits of detection for commercial ELISA, we analyzed female T samples using LC–MS/MS, which provides a more precise measurement of T. We then analyzed values using a *z*‐score transformation, since male and female raw values could not be directly compared. In future studies, all T samples could benefit from LC–MS/MS laboratory analysis, but this was cost prohibitive in the present study. Finally, there were some logistical considerations of the expedition itself that shaped the statistical analyses; specifically, since later sections were ranked more difficult than earlier sections, due to collinearity, section difficulty was excluded in models looking at energetic variables (i.e., %MVPA and AEE).

## 5 Conclusion

These results support the idea of physiological acclimatization via changes in baseline T and T reactivity across the course length, as well as the changing relationship of CORT and T reactivity with physical activity intensity and AEE. Given divergence in patterns connecting endocrine and energetic outcomes between males and females, this may point to a differing relationship between hormonal shifts and metabolic outcomes during acclimatization between sexes. Hormones such as CORT and T have important metabolic roles and are likely critical to acclimatization, contributing to energy availability and body composition changes. However, in field settings, particularly when individuals are in constrained social groups experiencing a novel environment under intensive energetic stress, neuroendocrine shifts only partially reflect energetic pressures, as we see here. Given that biological sex includes overlap between phenotypes and is difficult to untangle from gender identity and contextual gender roles (DuBois and Shattuck‐Heidorn 2021; van Anders and Watson 2006), hormonal shifts not explained by physical activity or energy expenditure could be linked to other interactions, including those from the social environment. As such, these sex differences do not reify a dichotomous outcome so much as they demonstrate critical variation in acclimatization patterns.

These responses may have implications for understanding how humans adjust to energetically intensive environments, particularly when experienced for the first time. This research critically informs how CORT and T response shifts across a relatively short period of time as individuals physiologically adjust to their environment and demonstrates how HPA and HPG activation and energetic outcomes, including physical activity and AEE, are related during the acclimatization process.

## Author Contributions

**Mallika S. Sarma:** conceptualization, investigation, funding acquisition, writing – original draft, methodology, visualization, writing – review and editing, formal analysis, project administration, data curation. **Cara J. Ocobock:** conceptualization, writing – original draft, methodology, writing – review and editing, formal analysis, resources. **Shannon Rochelle:** writing – review and editing, project administration, resources, data curation, methodology. **Sarah Martin:** methodology, writing – review and editing, project administration, data curation, resources. **Shalender Bhasin:** writing – review and editing, formal analysis, resources. **Clare Super:** writing – review and editing, visualization, data curation. **S. Finn Warrick:** visualization, data curation, writing – review and editing. **Lee T. Gettler:** conceptualization, funding acquisition, writing – original draft, methodology, writing – review and editing, formal analysis, project administration, resources, supervision.

## Funding

This work was supported by the National Science Foundation, DDRIG: #1847850 and Philanthropic Educational Organization (P.E.O) Scholar Award.

## Disclosure

No AI tools were used at any stage of manuscript preparation.

## Supporting information


**Data S1:** ajpa70360‐sup‐0001‐Supinfo.docx. **Supporting Information 1:** Energy expenditure equations.
**Supporting Information: Table 1:** Medication use.
**Supporting Information: Table 2** Linear mixed models predicting male CORT & T from hormone‐relevant medication^.
**Supporting Information: Table 3** Linear mixed models predicting female CORT & T from contraceptive medication use.
**Supporting Information: 2** Menstrual Data Analysis.
**Supporting Information: Table 4** Linear mixed models predicting female CORT from menstrual cycle phase.
**Supporting Information: Table 5** Linear mixed models predicting female T from menstrual cycle phase.
**Supporting Information: Table 6** Linear mixed models predicting CORT from time on course.

## Data Availability

The data that support the findings of this study are openly available in GitHub at https://github.com/habblab/nolsexpedition.
