# Supplementary material for: Changes in Cortisol and Testosterone During Acclimatization and the Relationship to Energetic Outcomes
Source: Am J Biol Anthropol. 2026 Oct 10;191(2):e70360. doi: 10.1002/ajpa.70360 (PMC13653219; doi:10.1002/ajpa.70360)
Supplement: Supplementary file 1 [file AJPA-191-e70360-s001.docx]

**Supplement 1.**

**ENERGY EXPENDITURE EQUATIONS**

Freedson VM3 Combination (’11)

*if VMCPM > 2453*
*Kcals/min= 0.001064×VM + 0.087512(BM) - 5.500229*

*where*
***VM*** *=* [*Vector Magnitude*](https://actigraph.desk.com/customer/en/portal/articles/2515527-what-is-vm-vector-magnitude-) *(sqrt((Axis 1)^2+(Axis 2)^2+(Axis 3)^2])* 
***VMCPM*** *= Vector Magnitude Counts per Minute*
***BM*** *= Body Mass in kg*

*if VMCPM < 2453*
*Kcals/min=CPM×0.0000191×BM*
*where*
***VM*** *=* [*Vector Magnitude*](https://actigraph.desk.com/customer/en/portal/articles/2515527-what-is-vm-vector-magnitude-) *(sqrt((Axis 1)^2+(Axis 2)^2+(Axis 3)^2])*
***VMCPM*** *= Vector Magnitude Counts per Minute*
***CPM*** *= Counts per Minute*
***BM*** *= Body Mass in kg*

**ACTIVITY INTENSITY CUT-POINTS**

Light: 0 – 2690 CPM

Moderate: 2691 – 6166 CPM

Vigorous: 6167 – 9642 CPM

Very vigorous: 9643 - ∞

*Suppl Table 1.*

|  | **Section 1** | **Section 2** | **Section 3** | **Section 4** |
| --- | --- | --- | --- | --- |
| **COURSE A** | hike | climb | canoe | canyon |
| **COURSE B** | hike | canoe | climb | canyon |
| **COURSE C** | mountaineering | kayak | climb | canyon |
| **COURSE D** | mountaineering | climb | kayak | canyon |
| **COURSE E** | hike | climb | canyon | winter |
| **COURSE F** | hike | canyon | climb | winter |
| **COURSE G** | hike | climb | canyon | winter |
| **COURSE H** | hike | climb | canyon | winter |
| **COURSE I** | wilderness EMT | canyon | climb | winter |
| **COURSE J** | wilderness EMT | climb | canyon | winter |
| High physical difficulty = hike, canyon, mountaineering, winter  Low physical difficulty = climb, canoe, wilderness EMT | | | | |

| Supplemental Table 1. Medication Use | | | | | | |
| --- | --- | --- | --- | --- | --- | --- |
|  |  | **Male** | |  | **Female** | |
|  | **Medication Type** | **Number** | **Percent** |  | **Number** | **Percent** |
| **Hormone-relevant Medications** | **Contraceptive (IUD/oral/implant/unspecified)*** | 0 | 0.0 |  | 14 | 35.9 |
|  | **Non-contraceptive Medications with Hormonal Effects*** | 5 | 14.7 |  | 5 | 12.8 |
|  | Antidepressant (SSRI & Others)* | 3 | 8.8 |  | 6 | 15.4 |
|  | ADHD medication* | 3 | 8.8 |  | 2 | 5.1 |
|  | Thyroid medication* | 1 | 2.9 |  | 0 | 0.0 |
|  | Anti-androgenic* | 0 | 0.0 |  | 1 | 2.6 |
| **Other reported medications** | Anti-viral | 1 | 2.9 |  | 2 | 5.1 |
|  | Allergy medication | 2 | 5.9 |  | 4 | 10.3 |
|  | Antibiotics | 0 | 0.0 |  | 0 | 0.0 |
|  | Acetaminophen & NSAIDs | 3 | 8.8 |  | 5 | 12.8 |
|  | OTC topical | 1 | 2.9 |  | 0 | 0.0 |
|  | Vitamins | 1 | 2.9 |  | 4 | 10.3 |
|  | Antifungal | 0 | 0.0 |  | 2 | 5.1 |
|  | Inhaler | 1 | 2.9 |  | 4 | 10.3 |
|  | Anti-epileptic medication | 1 | 2.9 |  | 1 | 2.6 |
|  | Anti-acne medication | 0 | 0.0 |  | 1 | 2.6 |
|  | Eye Drops | 0 | 0.0 |  | 2 | 5.1 |
|  | Other | 0 | 0.0 |  | 4 | 10.3 |
|  | **Any Medication** | 11 | 32.4 |  | 28 | 71.7 |
|  | **Multiple Medications**** | 5 | 14.7 |  | 13 | 33.3 |
| *For these medications, there is some evidence or plausible physiological pathways for an effect on CORT and/or T.  **For females, 74% used a contraceptive and some other kind of medication | | | | | | |

Females using contraceptives are overrepresented in the multiple medication category – as noted in the table above, the majority of females (74%) reporting hormone-relevant medication (including contraceptives) were using a contraceptive in addition to other hormone-relevant medication.

**Suppl Table 2. Linear mixed models predicting male CORT & T from hormone-relevant medication^**

The values below represent the linear mixed model results for hormone-relevant medication in fully-adjusted models across all four hormonal outcomes.

|  | *b* | 95% CI | *p* |
| --- | --- | --- | --- |
| Baseline CORT^a^ | -0.27 | -0.58, 0.03 | 0.08 |
| CORT reactivity^b^ | 32.58 | -8.87, 74.03 | 0.12 |
| Baseline T^c^ | -0.21 | -0.42, 0.002 | 0.05 |
| T reactivity^d^ | -8.25 | -28.66, 12.16 | 0.43 |

*Note*: *b* = fixed-effects coefficient; CI = confidence interval

* *p*<0.05; ** *p*<0.01; *** *p*<0.001

^a^n= 78; adjusted for baseline sampling time, age, and body fat, grouped by course and id; ^b^n= 42; adjusted for reactivity sampling time, reactivity time range, age, and body fat, grouped by course and id; ^c^n= 75; adjusted for baseline CORT, baseline sampling time, age, and body fat, grouped by course and id; ^d^n= 40; adjusted for baseline CORT, reactivity sampling time, reactivity time range, age, and body fat, grouped by course and id

^evidence of effect on CORT and/or T. See Supplemental Table 1.

**Suppl Table 3. Linear mixed models predicting female CORT & T from contraceptive medication use**

The values below represent the linear mixed model results for contraceptive medication in fully-adjusted models across all four hormonal outcomes.

|  | *b* | 95% CI | *p* |
| --- | --- | --- | --- |
| Baseline CORT^a^ | 0.26 | -0.02, 0.54 | 0.07 |
| CORT reactivity^b^ | -3.31 | -22.79, 16.16 | 0.74 |
| Baseline T^c^ | -0.64 | -1.32, 0.042 | 0.07 |
| T reactivity^d^ | -18.03 | -191.89, 155.82 | 0.84 |

*Note*: *b* = fixed-effects coefficient; CI = confidence interval

* *p*<0.05; ** *p*<0.01; *** *p*<0.001

^a^n= 99; adjusted for baseline sampling time, age, and body fat, grouped by course and id; ^b^n= 61; adjusted for reactivity sampling time, reactivity time range, age, and body fat, grouped by course and id; ^c^n= 93; adjusted for baseline CORT, baseline sampling time, age, and body fat, grouped by course and id; ^d^n= 56; adjusted for baseline CORT, reactivity sampling time, reactivity time range, age, and body fat, grouped by course and id.

Of the 19 females reporting taking medication, 14 reported some kind of contraceptive and only 5 reported non-contraceptive medication with known hormonal impacts; the direction of findings did not change with the inclusion of all medication so for females we specified for contraceptives specifically.

**Supplement 2. Menstrual Data Analysis**

Menstrual data was self-reported by each participant at each checkpoint. Participants recorded the first day of their last period (i.e., menstruation start date), and menstrual phase was calculated by subtracting menstruation start date from the sampling collection date to estimate the number of days into the menstrual cycle participants were in. Following calculation protocol by van Anders and colleagues (van Anders et al., 2014), if the estimated cycle time was less than **less than** 14 days, it was categorized as Luteal Phase, if **between** 15-16 days, it was categorized as Ovulatory Phase, and **more** than 16 days before the sampling date, it was categorized as Follicular Phase. For reported dates that suggested greater than 50 days into a cycle, phase was categorized as “irregular cycling” (Bull et al., 2019). For missing data, but with other menstrual cycle data points throughout the study, phase was categorized as “missing data but reported menstruating”.

**References**

Bull, J. R., Rowland, S. P., Scherwitzl, E. B., Scherwitzl, R., Danielsson, K. G., & Harper, J. (2019). Real-world menstrual cycle characteristics of more than 600,000 menstrual cycles. Npj Digital Medicine, 2(1), 83. <https://doi.org/10.1038/s41746-019-0152-7>

van Anders, S. M. V., Goldey, K. L., & Bell, S. N. (2014). Measurement of Testosterone in Human Sexuality Research: Methodological Considerations. *Arch Sex Behav*, *43*, 231–250. https://doi.org/10.1007/s10508-013-0123-z

**Suppl Table 4. Linear mixed models predicting female CORT from menstrual cycle phase**

The values below represent the linear mixed model results for contraceptive medication in fully-adjusted models across all four hormonal outcomes.

|  |  | ***b*** | **95% CI** | ***p*** |
| --- | --- | --- | --- | --- |
| **Baseline CORT^a^** | Luteal phase | -0.07 | -0.45, 0.32 | 0.735 |
|  | Ovulatory phase | -0.30 | -1.20, 0.61 | 0.522 |
|  | Follicular phase | -0.13 | -.056, 0.31 | 0.571 |
|  | Menstruating | -0.14 | -0.92, 0.64 | 0.726 |
|  | Irregular cycling (> 50 days) | -0.09 | -0.62, 0.44 | 0.740 |
| **CORT reactivity^b^** | Luteal phase | 1.16 | -27.29, 29.61 | 0.936 |
|  | Ovulatory phase | -17.02 | -88.78, 54.73 | 0.642 |
|  | Follicular phase | -7.59 | -37.33, 22.16 | 0.617 |
|  | Menstruating | -18.90 | -73.78, 35.98 | 0.500 |
|  | Irregular cycling (> 50 days) | -15.61 | -50.40, 19.182 | 0.379 |

*Note*: *b* = fixed-effects coefficient; CI = confidence interval

* *p*<0.05; ** *p*<0.01; *** *p*<0.001

^a^n= 79; adjusted for baseline sampling time, age, and body fat, grouped by course and id; ^b^n= 47; adjusted for reactivity sampling time, reactivity time range, age, and body fat, grouped by course and id;

**Suppl Table 5. Linear mixed models predicting female T from menstrual cycle phase**

|  |  | **b** | **95% CI** |  | ***p*** |
| --- | --- | --- | --- | --- | --- |
| **Baseline T^a^** | Luteal phase | -0.44 | -1.39, 0.52 |  | 0.368 |
|  | Ovulatory phase | 0.72 | -1.11, 2.54 |  | 0.441 |
|  | Follicular phase | 0.60 | -0.42, 1.62 |  | 0.247 |
|  | Menstruating | -1.02 | -2.66, 0.62 |  | 0.223 |
|  | Irregular cycling (> 50 days) | 0.27 | -1.04, 1.57 |  | 0.688 |
| **T reactivity^b^** | Luteal phase | 182.67 | -75.84 441.18 |  | 0.166 |
|  | Ovulatory phase | -237.93 | -835.58, 359.72 |  | 0.435 |
|  | Follicular phase | -20.13 | -292.66, 252.41 |  | 0.885 |
|  | Menstruating | -220.96 | -686.89, 244.97 |  | 0.353 |
|  | Irregular cycling (> 50 days) | 138.24 | -170.73, 447.20 |  | 0.381 |

*Note*: *b* = fixed-effects coefficient; CI = confidence interval

* *p*<0.05; ** *p*<0.01; *** *p*<0.001

^a^n= 74; adjusted for baseline CORT, baseline sampling time, age, and body fat, grouped by course and id; ^b^n= 44; adjusted for baseline CORT, reactivity sampling time, reactivity time range, age, and body fat, grouped by course and id

| **Suppl. Table 6. Linear mixed models predicting CORT from time on course** | | | | | | | | | | | | | | | |
| --- | --- | --- | --- | --- | --- | --- | --- | --- | --- | --- | --- | --- | --- | --- | --- |
|  | ***Baseline logged CORT*** | | | | | | |  | ***CORT Reactivity*** | | | | | | |
|  | *male^a^* | | |  | *female^b^* | | |  | *male^c^* | | |  | *female^d^* | | |
|  | ***b*** | **95% CI** | **p** |  | ***b*** | **95% CI** | **p** |  | ***b*** | **95% CI** | **p** |  | ***b*** | **95% CI** | **p** |
| **Main Effects** |  |  |  |  |  |  |  |  |  |  |  |  |  |  |  |
| timepoint |  |  |  |  |  |  |  |  |  |  |  |  |  |  |  |
| section 2 | 0.15 | -0.30, 0.60 | 0.517 |  | 0.38 | -0.07, 0.84 | 0.095 |  | 45.05 | -22.57, 112.66 | 0.192 |  | -30.55 | -79.03, 17.92 | 0.217 |
| section 3 | -0.04 | -0.43, 0.34 | 0.832 |  | 0.12 | -0.27, 0.50 | 0.551 |  | 2.62 | -52.40, 57.64 | 0.926 |  | -18.76 | -54.78, 17.26 | 0.307 |
| section 4 | -0.18 | -0.52, 0.16 | 0.301 |  | -0.05 | -0.39, 0.29 | 0.757 |  | 0.92 | -50.00, 51.84 | 0.972 |  | -3.72 | -33.30, 25.87 | 0.805 |
| **Covariates** |  |  |  |  |  |  |  |  |  |  |  |  |  |  |  |
| baseline sampling time | **-0.18** | **-0.23, -0.14** | **<0.001** |  | **-0.12** | **-0.17, -0.08** | **<0.001** |  |  |  |  |  |  |  |  |
| reactivity sampling time | - | - |  |  | - | - |  |  | -2.31 | -7.06, 2.43 | 0.339 |  | 0.71 | -2.13, 3.56 | 0.623 |
| reactivity time range | - | - |  |  | - | - |  |  | **-143.20** | **-240.70, -45.70** | **0.004** |  | **-71.26** | **-126.40, -16.13** | **0.011** |
| age | -0.01 | -0.03, 0.01 | 0.250 |  | -0.01 | -0.03, 0.02 | 0.530 |  | 1.23 | -1.09, 3.55 | 0.300 |  | 1.48 | **-0.08, 3.04** | 0.063 |
| body fat | **4.83** | **1.36, 8.30** | **0.006** |  | **-3.01** | **-5.11, -0.91** | **0.005** |  | 230.18 | -128.75, 589.12 | 0.209 |  | 3.34 | -143.76, 150.45 | 0.964 |
| section difficulty | 0.32 | -0.02, 0.66 | 0.067 |  | **0.48** | **0.12, 0.85** | 0.009 |  | 14.58 | -32.47, 61.62 | 0.544 |  | -18.18 | -51.17, 14.82 | 0.280 |
| medications^e^ | -0.27 | -0.57, 0.03 | 0.076 |  | **0.26** | **-0.01, 0.53** | 0.061 |  |  |  |  |  |  |  |  |
| ^a^obs = 78; ^b^obs = 99; ^c^obs = 42; ^d^obs = 61; ^e^male medications include all hormone-relevant medication and female medications are specifically contraceptive medications | | | | | | | | | | | | | | | |
